# The role of high-resolution manometry in a resource challenged service

**DOI:** 10.4102/jcmsa.v3i1.113

**Published:** 2025-02-13

**Authors:** Mohammed A. Parker, Mogamad S. Gabriel, Muhammed S. Moolla, Christoffel J. van Rensburg, Desiree L. Moodley, Wesley P. du Plessis, Ahmad A. Abdelsalem

**Affiliations:** 1 Division of Gastroenterology and Hepatology, Department of Medicine, Faculty of Medicine and Health Sciences, Stellenbosch University, Tygerberg Hospital, Cape Town, South Africa; 2 Division of Pulmonology, Department of Medicine, Faculty of Medicine and Health Sciences, Stellenbosch University, Tygerberg Hospital, Cape Town, South Africa

**Keywords:** high-resolution manometry, oesophageal motility disorders, Chicago classification, South Africa, ineffective oesophageal motility, achalasia

## Abstract

**Background:**

Oesophageal high-resolution manometry (HRM) is commonly used in the evaluation of patients presenting with upper gastrointestinal tract symptoms where primary oesophageal motility disorders are suspected. At our hospital, one doctor can perform and interpret HRM, with the hospital serving a population of approximately 3 million people. There is a paucity of data on HRM findings in South Africa.

**Methods:**

This study included data from patients that underwent HRM between January 2018 and January 2023, using the hospitals electronic note-keeping system to access records. High-resolution manometry was performed for all patients using a water state catheter with 24 pressure channels. The Chicago classification version 3 was used for reporting.

**Results:**

A total of 210 patients were included in this study. The average age of patients included was 50 years (standard deviation [s.d.] ± 15.2) old with a female predominance (*n* = 149, 71.0%). Indications included gastroesophageal reflux disease (GORD) (65.7%), refractory heartburn (62.4%), dysphagia (37.6%), non-cardiac chest pain (16.7%), pre-procedurally (5.7%) and post-procedurally (14.8%). Many primary oesophageal motility disorders were observed, including ineffective oesophageal motility (20%), achalasia (11.4%), absent contractility (4.3%), oesophagogastric junction outflow obstruction (1.9%) and jackhammer oesophagus (1%). Normal findings accounted for 61%. The most common type of achalasia was type 1 (*n* = 16, 66%).

**Conclusion:**

This study, being the first of its kind in South Africa, highlights the role of HRM in the diagnosis of primary oesophageal motility disorders with ineffective oesophageal motility being the most common pathology and GORD being the most common indication for HRM. Achalasia type 1 was the common type diagnosed.

**Contribution:**

HRM, despite its scarce availability, remains an important diagnostic tool in evaluating gastrointestinal pathologies.

## Introduction

Oesophageal high-resolution manometry (HRM) is commonly used in the evaluation of patients presenting with upper gastrointestinal tract symptoms where primary oesophageal motility disorders are suspected.^1^ Oesophageal manometry assesses the motility by measuring the amplitude of the contractile events within the oesophagus and its sphincters in relation to time.^2^ The Chicago classification is the internationally preferred method of interpreting the results of HRM.^3,4^

The classification categorises oesophageal motility disorders via an algorithmic scheme using metrics from HRM.^5^ By using the Chicago classification, HRM has superior inter-rater agreement with high accuracy in reaching the diagnosis, even for non-experts and is easier to learn.^6,7^ Currently, the latest version is the fourth version.^5^

Common symptoms include dysphagia, heartburn, chest pain and regurgitation.^8^ Achalasia, oesophageal junction outflow tract obstruction, ineffective motility and absent contractility are some of the diagnoses that may be made by HRM.^3^

The aim of our study was to retrospectively assess the indications, as well as pathology found at HRM over a 5-year period at Tygerberg Hospital in the Western Cape South Africa. We also assessed achalasia and the different types diagnosed at our hospital.

## Research methods and design

A retrospective descriptive study was performed using Tygerberg Hospital’s electronic note-keeping system. The electronic folders of all patients that had a HRM procedure performed during the study period were considered for inclusion. All necessary data needed from the folders were extracted and entered into a password-protected data sheet.

Data analysis was performed using VassarStats (available at http://vassarstats.net). Categorical data were described according to number and proportion. Continuous data with a normal distribution were described using means and standard deviations (s.d.), whereas non-normal data were described using medians and interquartile ranges (IQRs).

The protocol of our unit is to perform HRM on all patients with persistent bothersome upper gastrointestinal symptoms that have had an inconclusive endoscopy, inconclusive barium swallow, non-response to anti-secretory therapy and a primary motility disorder is suspected.

An oesophageal HRM procedure was performed for all patients (medical measurement systems [MMS] Laborie device), using a water state catheter with 24 pressure channels. Patients were fasted for 6 h – 8 h prior and informed to stop any anticholinergic drugs, nitrates, calcium channel blockers and prokinetic drugs, for a minimum of 72 h before the procedure. Patients were informed to stop proton pump inhibitor therapy at least 2 weeks prior to their procedure.

After catheter calibration and the application of a topical anaesthetic to the patient’s nostril, the HRM catheter was placed transnasally and positioned with the pressure sensors spanning a length extending from the hypopharynx, through the oesophagus, to 3 cm to 5 cm within the stomach. This was performed while the patient was sitting up, after which the patient was made to lie flat. Confirmation of correct positioning in the stomach was confirmed by recognition of the presence of the pressure inversion point (PIP), which is the point at which the inspiration-associated negative intrathoracic pressure inverts to the positive intra-abdominal pressure. These changes were augmented by taking three deep breaths.

Ten supine liquid swallows of 5 mL were performed initially with a break of at least 20 s in between. Thereafter, the patient was asked to stop swallowing for at least 30 s to assess for resting integrated relaxation pressure (IRP). Following this, patients performed multiple rapid swallows: 5 swallows of 2 mL at 2 s – 3 s intervals. This assesses peristaltic reserve. Finally, patients were asked to rapidly drink 200 mL of water, as part of the rapid drinking challenge. After this, the procedure was stopped and the catheter removed.

The results of the HRM findings were interpreted using the Chicago version 3.

### Ethical considerations

Ethical clearance to conduct this study was obtained from the Stellenbosch University Faculty of Health Sciences Human Research Ethics Committee (No. S23/06/138).

## Results

A total of 213 patients were identified who underwent HRM during the 5-year study period. Three were less than 18 years old and were excluded. The remaining 210 patients were included in the analysis. The average age of patients included was 50 years (s.d. 15, 2) old with a female predominance (*n* = 149,71%). Most patients included in the study had upper gastrointestinal symptoms with a minority being investigated either as part of their preoperative or postoperative assessments. The most common indications were gastroesophageal reflux disease (GORD) (65.7%) and refractory heartburn (62.4%) with the least common being preoperative (5.7%) (see Table 1). Most of the HRM findings were normal (61%). Hiatus hernia was found in 41.9% of our patients. There was a female (*n* = 69, 68.4%) predominance.

**TABLE 1:** Indications for high-resolution manometry.

Indication for HRM	*n*	%
GORD	138	65.7
Dysphagia	79	37.6
Non-cardiac chest pain	35	16.7
Refractory heartburn	131	62.4
Pre-procedure	12	5.7
Post-procedure	31	14.8

HRM, high-resolution manometry; GORD, gastroesophageal reflux disease.

Patients who had HRM pre-operatively and post-operatively accounted for 20.5% of all study participants. Pre-procedurally, the only indication identified was as part of anti-reflux surgery work up. Post-procedurally, Heller myotomy accounted for 10 patients, post-balloon dilatation of achalasia for four patients and Nissen fundoplication for 17 patients.

Many primary oesophageal motility disorders were observed including ineffective oesophageal motility (IEM), achalasia, absent contractility, OGJ outflow obstruction, jackhammer oesophagus and normal findings (see Table 2). The most common finding was IEM (20%), with the least common being jackhammer oesophagus (1%). The most common type of achalasia seen was type 1, with no type 3 achalasia being diagnosed.

**TABLE 2:** Diagnostic findings of high-resolution manometry.

Diagnosis	*n*	%
Normal	129	61.0
Achalasia	24	11.4
**Achalasia type**		
1	16	66.7
2	8	33.3
Ineffective oesophageal motility	42	20.0
Absent contractility	9	4.3
OGJ outflow obstruction	4	1.9
Jackhammer oesophagus	2	1.0

OGJ, oesophago-gastric junction.

## Discussion

This study describes primary oesophageal motility disorders as well as indications for HRM in South Africa.

There is a paucity of data on HRM in South Africa, and our findings can only be compared to those found in other countries. The average age of our patients was similar to a study conducted in Pakistan (48 years old).^9^ Our study differed from studies performed in Egypt and Pakistan where the most common indication was dysphagia 41.3% and 75.2%, respectively.^8,9^ A possible explanation for this may be that in our hospital, patients presenting with dysphagia are generally investigated firstly radiologically, with a barium oesophagogram and then secondly, with upper gastrointestinal endoscopy while being on twice daily proton pump inhibitors. The diagnosis of pathology at HRM was less compared to studies performed in Egypt and Pakistan where our reported pathological findings were 39% compared to 68.8% and 79.2%, respectively.^8,9^ Causes for this may be multifactorial; population-based factors including genetics, diet, age and ethnicity. Patients may also have presented with advanced pathology in the other studies. A significant number of HRM studies (61%) in our population were normal; however, strict criteria as mentioned earlier were adhered to prior to performing the investigation. Normal HRM also forms a crucial part of the diagnosis of functional oesophageal disorders.^10^

The most common finding on HRM was IEM (20%). This is close to the reported international prevalence of 30%.^11^ Ineffective oesophageal motility is not associated with any specific disease and can be found in healthy individuals without symptoms.^11^

The diagnosis of achalasia differed most when compared to studies conducted in other countries with our reported percentage of 11.9% being far fewer than 35.9% and 20.9% reported in Pakistan and Egypt, respectively.^8,9^ A possible explanation may be that during the time that our study was conducted, it was the coronavirus disease 2019 (COVID-19) pandemic, which resulted in less number of patients seeking healthcare for upper gastrointestinal symptoms, as well as our services being redirected towards emergency care. The pandemic affected regular services for 2 years of the 5 years that our study was conducted in. Our centre is one of the two servicing 7 million people at a tertiary level across the province, where many of our patients are from lower socio-economic backgrounds and it is very hard for them to travel to our centre. First and follow-up visits are often missed by our patients for this important reason.

This study, being the first of its kind in South Africa, describes HRM findings and common indications. It has identified and quantified the various findings over a 5-year period and can serve as a reference for further studies performed in South Africa in terms of presenting symptoms as well as HRM findings. Further studies may also include the types of achalasia as well as management outcomes of which there is no published data in South Africa.

Primary oesophageal motility disorders are being diagnosed in our population and interventions to aid in identifying these patients should be taken into consideration. Outreach programmes to secondary-level facilities to educate junior and non-specialised healthcare workers is a realistic and viable way of achieving this goal. Outreach would also facilitate better referral pathways once patients with a suspected oesophageal motility disorder are identified.

Our study does have some limitations. The study was retrospective. The population number was small, and the study was conducted in a single centre. We were unable to do subgroup analysis in this study because of the small size of the subgroups.

## Conclusion

This study, being the first of its kind in South Africa, describes HRM findings with IEM being the most common pathology, and GORD being the most common indication for HRM. Achalasia type 1 was the most common type diagnosed in our population.
